# Factors Influencing the Purchase Intention for Online Health Popular Science Information Based on the Health Belief Model

**DOI:** 10.3390/bs13080693

**Published:** 2023-08-20

**Authors:** Jingfang Liu, Shiqi Wang

**Affiliations:** School of Management, Shanghai University, Shanghai 201800, China; jingfangliu@shu.edu.cn

**Keywords:** online paid health information, purchase intention, knowledge payment, health belief model

## Abstract

There is a growing demand for health popular science information from the public. Online paid health popular science information provides a new channel for the public to obtain health popular science information and can meet users’ demands for high-quality health popular science information. In order to improve the popularity of online paid health popular science information, it is urgent to investigate the factors influencing users’ intention to pay for online health popular science information. Paid online health popular science information can provide users with higher-quality health knowledge, while monetary compensation can incentivize publishers to create and promote the sustainability of social media platforms. Therefore, paid online health popular science information is important for readers, creators, and platforms. Therefore, this study investigates the factors influencing users’ intentions to purchase online health popular science information based on the health belief model (HBM). Our research data were obtained by a questionnaire and empirically analyzed by SmartPLS structural equation modeling. The results of this study indicate that the greater the perceived susceptibility, severity, and irreplaceability of health problems, the greater the intention of users to purchase health information when they read the summary portion of paid online health popular science information. And the higher the perceived risk and the more unreasonable the price of the online health popular science information, the lower the intention to purchase. Moreover, both the perceived susceptibility and perceived severity significantly attenuated the negative impacts of perceived health popular science information risk and perceived price unreasonableness on users’ intentions to purchase online health popular science information. This study not only enriches and extends the application of the health belief model, but also has important positive implications for the development of online paid health popular science information.

## 1. Introduction

In recent years, with the development of Internet technology and the public’s demand for high-quality information, online information payment has gradually become an emerging trend [1]. A series of paid reading, paid Q and As, paid courses, paid subscriptions, and other kinds of paid information products have emerged and attracted many consumers [2]. Users can obtain more valuable and higher-quality information by paying for information [3].

With the increase of public health awareness and health literacy, online paid information is gradually appearing in the online health field [4]. Paid health popular science information is beginning to appear on the Internet to meet users’ needs for higher-quality health popular science content. Health popular science is the introduction of professional health popular science knowledge to the public in an easy to understand way [5]. In addition, many users use the health popular science information obtained through the Internet as an important reference basis for their health decisions [6,7,8]. Moreover, health popular science information itself has a certain degree of medical expertise, and it is difficult for ordinary users to recognize its accuracy [9]. Paid health information can provide users with more professional, comprehensive, and relevant health knowledge [3]. At present, MicroBlog, Zhihu, and other major social media platforms have paid health information sections to provide users with high-quality health popular science information in the forms of graphics and videos [10]. Users can see the titles and partial summaries of the paid health information, judge the value of the limited content, and then purchase the complete health information through the platform. Online paid health information not only enables social media platforms to gain more revenue for sustainable development, but also inspires contributors to publish more high-quality health information. Therefore, online paid health information is of great importance to ordinary users, creators, and platforms [11].

Although online paid health popular science information can provide higher-quality health knowledge, the popularity of paid online health popular science information is still low. The number of purchases of online paid health popular science information is much lower than the number of readings of free health popular science information [12]. Most users are more accustomed to obtaining health popular science information through free Internet channels. And paid online health information is a very important topic of paid content in the field of paid knowledge. Paid online health information can provide the public with richer and more professional health knowledge. And at the same time, it can motivate more medical professionals to publish higher-quality health information. In order to increase the popularity of online health popular science information and thus the purchase of paid health information, it is important to understand what types of paid health information motivate users to purchase them. Therefore, our research question aims to investigate the factors that influence users’ intentions to purchase online health popular science information.

In order to address the above questions, this study constructed a research model based on the health belief model to investigate the factors influencing users’ purchase intentions for online health popular science information. Health belief models are primarily currently used to explain people’s health-related behaviors. This model posits that people’s intentions to engage in certain health behaviors are influenced by the health beliefs that characterize conditioned perceptions [13]. The objective of this study is to investigate which psychological perception characteristics of users after reading the summaries of paid online health popular science information can motivate users to generate purchase intentions. We tested this research model by means of a questionnaire survey. In addition, we further investigated the moderating effects of two health belief variables, perceived susceptibility and perceived severity, on users’ intentions to purchase paid online health popular science information. Studies have been conducted to investigate the impact on purchase intentions, mainly from the perspective of online information quality and contributors [14,15], or focus on accessing health popular science information in the form of one-on-one paid consultations to investigate the factors influencing the intentions to pay [16]. Few studies have focused on this form of paid health popular science information in social media platforms and explored the factors influencing users’ intentions to purchase health popular science information. In addition, purchases of online health popular science information are currently low [12]. Only by knowing what characteristics of health popular science information users pay more attention to can creators be more targeted in their content writing, thus increasing the popularity of paid online health popular science information and thus the purchase volume. It is not known which factors will stimulate users’ intentions to purchase online health popular science information and which factors will inhibit them. Therefore, it is important to investigate the factors influencing users’ intentions to purchase online health popular science information.

## 2. Literature Review

### 2.1. Research Related to Factors Influencing Online Information Purchase Intention

Online information payment refers to the transformation of information into a tradable product or service that allows the recipient of the information to pay the information provider indirectly through some channel; in many of the literatures online, information payment is also referred to as online knowledge payment [17]. Social media platforms share parts of paid information to attract users and thus stimulate their intentions to purchase. Current studies have explored the impact on users’ intentions to purchase, mainly from the perspectives of information contributors and the characteristics of the information itself. From the perspectives of online information contributors, it has been found that reputation, the number of times of free online information sharing, whether there is real name authentication, and the experience and integrity of online information contributors can significantly affect users’ purchase intentions [18]. From the perspective of the characteristics of online information itself, previous studies have shown that the perceived quality of free content, the number of participants, price, and perceived value can all positively influence users’ purchase intentions [19]. In addition, peer influence can also have a significant impact on users’ online information payment behaviors, and studies have found that peers, especially opinion leaders, have a strong influence on users and can affect their final purchase intentions to a certain extent [20]. Other studies have explored the factors influencing online information purchase intention from the perspective of the convenience of the purchase channel and found that both perceived usefulness and perceived ease of use can positively influence purchase intention [21].

Based on the above literature review, we found that the factors influencing online information purchase intention are mainly information provider characteristics, perceived information quality, and information purchase characteristics. However, few scholars have studied the factors influencing the intention to purchase online information on health topics. Due to the information asymmetry between doctors and patients, the value users perceive from the limited content of paid health popular science information may have a more important influence on their purchase intentions [22]. Therefore, this study focuses on the topic of online health popular science information, incorporates features related to health issues, and investigates the factors influencing users’ intentions to pay for online health popular science information.

### 2.2. Research Related to Online Paid Health Information

With the improvement of public health literacy, people’s demands for the quality of health popular science information on the Internet is increasing, and different forms of paid health information are rapidly developing. Currently, the main forms of paid access to health information on the Internet are paid consultations, paid Q and A, paid courses, and paid health popular science information [23]. Studies have focused on access to online health information in the forms of paid consultations and paid Q and As. It has been shown that the online reputations of doctors and active sharing of free health information can motivate users to purchase online consultations in order to obtain online health information from them [24]. Conversely, health information privacy issues significantly and negatively affect users’ intentions to purchase health information through online consultations [25]. Other scholars focused on accessing online health information by purchasing online mental health services, and they found that a perceived e-word-of-mouth trustworthiness, e-health literacy, perceived competence, and perceived price can significantly influence users’ purchase intentions [16]. It has also been verified that personalized health information can be accessed through the purchase of online health Q and A services [26]. In addition, scholars have also explored the motivation of physicians to provide paid health popular science information from the perspectives of physicians and found that, in addition to compensation motivation, the professional motivation of physicians is also an important motivation [4]. Other scholars have focused on online health information searching behavior and found that only about one-third of adults who conducted health information searching via the Internet were able to successfully access the health information they wanted [27]. Another study explored the factors influencing the consumer acceptance of health information technology; self-efficacy, subjective norms, trust, and perceived behavioral control were found to significantly and positively influence the consumer reception of health information technology [28]. In addition, there are scholars focusing on online social networks to explore the influencing factors for users to generate specific information behaviors. One study found that users’ intentions to comment and retweet negative CSR news on social media platforms are influenced by information usefulness, corporate image, and attitudes toward information behavior [29]. It has also been found that influential members of online social networks may often be more likely to generate negative electronic word-of-mouth [30].

In summary, the current research related to paid access to online health information has focused on one-to-one interaction processes such as paid consultations or paid Q and As. Few studies have focused on one-to-many paid health information on social media platforms. Paid consultations and paid Q and As are online health information that users acquire instantly by consulting with doctors and are not public in nature. Unlike the paid health popular science information on the social media platforms that this study focuses on, it is the health popular science information about disease, lifestyle, or dietary habits that the public has recently paid attention to, which is organized and refined by medical professionals or public health institutions and is open to all users of the platform and has a dissemination effect [3]. Users on the platform can see this paid health popular science information and choose whether to purchase it, according to their own health needs. Therefore, this study focuses on the paid access to online health information in the form of paid health popular science information published in one-to-many social media platforms and further investigates the factors influencing users’ intentions to purchase online paid health popular science information.

## 3. Theoretical Background and Research Hypothesis

### 3.1. Theoretical Background

The health belief model (HBM) was presented in the 1950s to explain personal health behaviors [31]. This model suggests that the intention to adopt proactive health behaviors relies more on individuals’ beliefs about specific conditions [13]. According to the health belief model, people’s adoption of health behaviors is determined to a large extent by their perceived susceptibility to disease, perception of the severity of the consequences of the disease, perceived benefits of the health behaviors in terms of threat reduction, and perceived barriers to adopting the negative implications of the health behaviors [31].

Currently, the HBM has been widely used to explain various health-related behaviors, including the intention to vaccinate with the COVID-19 vaccine [32,33], oral health behaviors [34], marine conservation [35], and factors influencing the intention to travel in the context of COVID-19 [36,37,38]. In addition to offline health-related preventive behaviors, the health belief model is gradually being applied to health services on the Internet [39]. For example, studies show that perceived susceptibility and perceived severity among health belief-related variables can significantly influence users to share as well as seek health popular science information on the Internet [40,41]. Studies have also explored the influences that promote users’ smartphone avoidance intentions, based on the HBM [42]. The health belief model is also a good explanation for adolescents’ Internet addiction behaviors [43].

In conclusion, the application of the HBM has evolved from the traditional adoption of disease prevention measures to general health-related behaviors. Although many studies have applied the HBM to health-related behaviors on the Internet, few studies have explained users’ online health popular science information purchase intentions based on this model. Therefore, this study investigates the factors influencing users’ intentions to purchase health popular science information on the Internet based on the HBM.

### 3.2. Research Hypothesis

The health belief model suggests that individuals’ beliefs about health risks predict the likelihood of adopting health-related behaviors [44]. With respect to a disease itself, the perceived risk for a health problem consists of two aspects: perceived susceptibility and perceived severity [45]. Once a person perceives the health risk of a particular health problem, he or she then forms beliefs about the possibility of health problems and the severity of the disease suffered. Studies have shown that both perceived susceptibility and perceived severity can motivate individuals to actively assess their health and take appropriate preventive measures [46]. Mou Jian et al. found that perceived severity and perceived susceptibility can significantly and positively influence users’ acceptance of health-related services on the Internet [47]. In this paper, we focus on users’ purchase intentions for online health popular science information and apply the HBM to the field of knowledge payment. Most of the online health-related research involving the variable of perceived risk is based on the risk perception of the disease itself. Many scholars have used perceived susceptibility and perceived severity in health belief models to measure the perceived risk of diseases. It has been found that consumers’ perceived susceptibility and perceived severity of common health problems can significantly stimulate their intentions to purchase healthy foods [48,49]. Moreover, two health beliefs based on perceived susceptibility and perceived severity of diseases can significantly and positively influence users’ disease-related preventive behaviors [50,51]. Based on the above literature review, we believe that in this research scenario, users may be further motivated to take preventive measures, i.e., purchase paid health popular science information, when they perceive greater susceptibility and severity from the health problems involved in paid health popular science information.

Therefore, in summary, we propose the following hypotheses:

**Hypothesis** **1** **(H1).** *Perceived susceptibility positively influences users’ purchase intentions for online health popular science information.*

**Hypothesis** **2** **(H2).** *Perceived severity positively influences users’ purchase intentions for online health popular science information.*

In addition, perceived information risk is one of the important information characteristics that influences user-related behaviors in research related to the field of information systems. Previous studies have shown that perceived risk is a key factor in the online purchasing environment, which refers to the possible negative consequences of purchasing a product or a service [52]. In such a virtual online environment based on content payment, many scholars have further refined the perceived risk. Studies have shown that information-based perceived risk is one of the uncertainties in e-commerce environments [53]. Other scholars have found that the credibility of the source of online information and the quality of information are important dimensions that influence perceived information risk and can significantly influence people’s online engagement during COVID-19 [54]. Based on the research context of this paper, we argue that health popular science information may further amplify users’ perceptions of information risk. Due to the importance of health issues to people, users will be more cautious when engaging in health-based content purchase behavior. In this study, to distinguish from perceived disease risk, we uniformly define perceived health popular science information-based risk as perceived health popular science information risk.

In summary, we formulate the following hypothesis:

**Hypothesis** **3** **(H3).** *Perceived health popular science information risk negatively affects users’ purchase intention for online health popular science information.*

The characteristic of perceived unique value attributed to the adoption of a certain behavior is defined in psychology as perceived irreplaceability [55]. Perceived irreplaceability is an individual’s perception that this behavior cannot be replaced by other behaviors, due to the unique value perceived by the individual. The benefits perceived by an individual while performing a certain behavior motivate him or her to focus more on that behavior [56]. In this paper, we focus on the intention to purchase health popular science information on the Internet, and because most of the health popular science information available on the Internet is currently free, the purchase of paid health popular science information has not yet reached a very significant value. Therefore, based on such a special market environment, we believe that users’ intentions to pay for online health popular science information is more likely to be stimulated only when they can perceive a unique value from paid health popular science information, which is not available from other free health popular science information.

According to the above formulation, we formulate the following hypothesis:

**Hypothesis** **4** **(H4).** *Perceived irreplaceability positively influences users’ purchase intentions for online health popular science information.*

As previous studies have shown, perceived price significantly affects purchase intention for online content [57,58]. In the field of marketing, pricing policies significantly affect consumers’ trust in retailers [59]. When consumers perceive that a product is reasonably priced, they are more inclined to purchase that product. This is also true in the field of paid content, where users may be less willing to pay for online health popular science information when they perceive it to be overpriced.

Based on this, we formulate the following hypothesis:

**Hypothesis** **5** **(H5).** *Perceived price unreasonableness negatively affects users’ purchase intentions for online health popular science information.*

Based on two variables of health beliefs, perceived susceptibility and perceived severity, this study further explored the moderating effect of these two health belief variables on the intention to pay for online health popular science information. The severity of disease has been shown to influence consumers’ perceptions of risk and subsequent behaviors [60]. They found that the severity of COVID-19 can moderate the negative implication of the perceived risks of purchasing digital food. Individuals’ perceived distance from the health problem can lead to different perceptions of risk [61]. For example, differences between regions in the severity of COVID-19 may affect customers’ emotions [62,63]. It has also been found that explanatory mindsets have opposite effects on the purchase intentions of customers in regions with high and low risks of the epidemic [64]. And perceived susceptibility and perceived severity have similar psychological perceptions in the general public. One study found that perceived susceptibility significantly moderated the effect of health education interventions on health screening behavior [65]. In summary, based on the above hypotheses, in this study we suggest that perceived susceptibility and perceived severity may attenuate the negative implications of perceived health popular science information risk and high price on users’ intentions to pay for online health popular science information. When users perceive that a health problem described in online health popular science information is more severe or more likely to be infectious and they perceive a higher risk of this disease, they will be more interested in knowing the preventive measures to deal with this disease risk, and their intention to pay for this health popular science information may be stronger, and then their concern for the risk and price of online health popular science information may be less.

In summary, we propose the following hypotheses:

**Hypothesis** **6a** **(H6a).** *Perceived susceptibility weakens the negative effect of perceived health popular science information risk on purchase intention for online health popular science information.*

**Hypothesis** **6b** **(H6b).** *Perceived susceptibility weakens the negative effect of perceived price unreasonableness on purchase intention for online health popular science information.*

**Hypothesis** **7a** **(H7a).** *Perceived severity weakens the negative effect of perceived health popular science information risk on purchase intention for online health popular science information.*

**Hypothesis** **7b** **(H7b).** *Perceived severity weakens the negative effect of perceived price unreasonableness on purchase intention for online health popular science information.*

**Figure 1:**
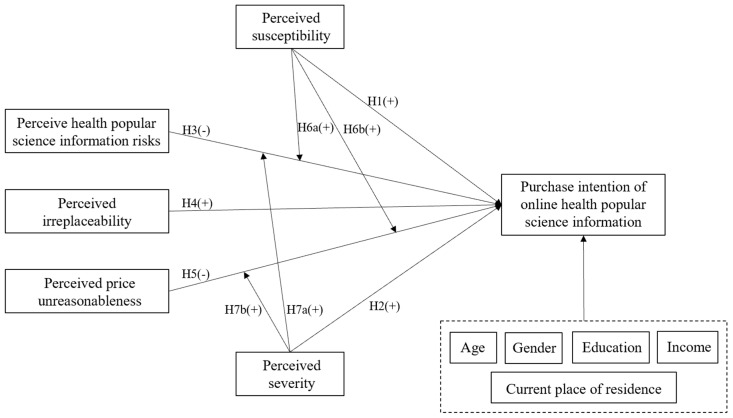
Research model.

In summary, this paper introduces health belief-related variables into the health knowledge payment scenario based on the HBM. And according to the characteristics of online paid health popular science information, perceived irreplaceability, perceived health popular science information risk, and price are added as independent variables. The moderating effects of perceived susceptibility and perceived severity are further explored. And the control variables include age, gender, education, income, and current city of residence. Finally, the study model is proposed, as shown in Figure 1.

## 4. Research Methodology

### 4.1. Scale Design

This study used a questionnaire to empirically analyze the proposed hypotheses. We designed a questionnaire on factors influencing users’ intentions to purchase online health science information. The questionnaire consisted of three sections: filtering items, demographic variables, and latent variables. The filtering item was a simple mathematical calculation question, “100 + 100 = ?” We randomly inserted a screening question into the questionnaire. If the screening question was answered incorrectly, it meant that this subject was not serious in answering the questionnaire [66]. And it is likely that the questionnaire was filled out randomly. Such questionnaire data do not have reference value. Therefore, in order to avoid this kind of questionnaire data from influencing the final conclusion of the study, all the questionnaire data with wrong answers to the math calculation questions were deleted in this study. The second section contained demographic variables, including age, gender, education, income, current city of residence, and whether they had purchased online health popular science information. The third section was the main body of the questionnaire, which was used to measure the latent variables involved in the research model. The latent variables included perceived susceptibility (PSU), perceived severity (PSE), perceived health popular science information risk (PHIR), perceived irreplaceability (PIRE), perceived price unreasonableness (PPUN), and intention to purchase online health popular science information (PI). Each latent variable was measured on multiple dimensions. The question items of the questionnaire for all the latent variables were obtained from relevant references. Based on the research scenario and the different research subjects, we made appropriate adjustments to the presentation. The questionnaire for this study was based on a 5-point Likert scale, ranging from strongly disagree to strongly agree. And the relevant latent variables and specific questionnaire items, as well as the reference sources for the items, are shown in Table 1.

### 4.2. Data Collection

This study created and distributed the questionnaire through a professional questionnaire platform, Credamo. The purpose of this questionnaire was introduced to the participants at the beginning of our questionnaire, and consent was obtained from the participants. Each participant will be rewarded with a certain amount of money. The questionnaire was collected from 11 February 2023 to 1 March 2023. And 654 questionnaires were collected. To guarantee the authenticity and the validity of the follow-up study, we excluded questionnaires with incorrect answers to the screening questions, short response times, and logically contradictory answers. Finally, after screening, 604 valid questionnaires were collected, and the effective recovery rate was 92.35%. This questionnaire survey first collected the demographic data of the participants in the first part of this questionnaire, which are listed in Table 2. Based on the data results, most of the participants were in the age group of 19–40 years old, and most of them had the highest education level of a bachelor’s degree. Most of participants had upper–middle income levels. 

## 5. Results

In this study, SmartPLS was used to analyze and validate the proposed hypotheses. Based on passing the validity and reliability tests, the study model was tested using partial least squares (PLS). It was shown that the partial least squares method is appropriate for studies with small and medium sample data [71]. Based on this, it is appropriate to choose the partial least squares method for the empirical analysis in this study.

### 5.1. Reliability and Validity Test

Reliability is an indicator of the accuracy and consistency of questionnaire scales. According to the summary of the previous literature, when the Cronbach’s α and the combined reliability (CR) exceed 0.7, it means the scale data results are acceptable and meet the reliability requirements [72]. According to the results in Table 3, we can find the CRs all exceed 0.7, the Cronbach’s α of each latent variable range from 0.711 to 0.872, and this study model has great internal consistency.

Validity includes content validity and structural validity. All the questions of the latent variables involved in this questionnaire were referred to in the previous related literature. Therefore, the questionnaire has great content validity. Moreover, the structural validity includes convergent validity and discriminant validity. In this study, the convergent validity and discriminant validity were analyzed separately in the validation factor analysis. According to the results in Table 3, the factor loadings of all the latent variables over 0.7 and the AVEs exceed 0.5, showing the model has great convergent validity. Discriminant validity means that the observed values can be distinguished when different methods are applied to measure different variables. And discriminant validity is tested by comparing the square root of the AVE and the correlation coefficient between these variables and other variables [73]. As shown in Table 4, the square root of the AVE of all the variables in this study is greater than the relevance between those variables and other variables. This result indicates the model of this research has great discriminant validity, and our measurement model is reliable.

### 5.2. Structural Model Inspection

#### 5.2.1. Main Effect Test

In this paper, main effects and moderating effects are analyzed separately in testing the structural model. Previous studies have concluded that a research model in the field of consumer behavior with an R2 exceeding 0.2 is an acceptable result [74]. The R2 of users’ online purchase intentions in the model of this study is 0.299, which means that the model fit is acceptable.

Before conducting the main effects test, we conducted partial least squares regression analysis on the control variables, and the results are shown in Table 5. We first recoded the control variables: age according to the 7 age groups from low to high, respectively, 1–7; 1 for female and 2 for male; 1–4 for education, according to lowest to highest; 1–6 for income, according to lowest to highest; and 1–5 for current city of residence from fifth-tier to first-tier cities, according to the city class classified by the First Financial Weekly. The regression results indicated that men are more likely to purchase online health popular science information compared to women. Income is positively correlated with the intention to pay for online health popular science information. People with relatively higher incomes are more likely to be willing to purchase online health popular science information.

We then conducted a main effects test, and the results are shown in Table 6. Perceived susceptibility was significantly positively correlated with purchase intention for online health popular science information (β = 0.101, *p* < 0.05); H1 was supported. Perceived severity positively affected purchase intention for online health popular science information (β = 0.112, *p* < 0.05); H2 was supported. Perceived health popular science information risk negatively affected purchase intention for online health popular science information (β = −0.211, *p* < 0.001); H3 was supported. Perceived irreplaceability was significantly positively correlated with purchase intention for online health popular science information (β = 0.148, *p* < 0.001); H4 was supported. Perceived price unreasonableness negatively influenced purchase intention for online health popular science information (β = −0.231, *p* < 0.001); H5 was supported.

#### 5.2.2. Moderating Effect Test

The moderating effects of two health belief variables, perceived susceptibility and perceived severity, are further explored in this paper. Perceived susceptibility and perceived severity interacted with perceived health popular science information risk and perceived price unreasonableness in the model, respectively, to examine the effect of the interaction term on users’ intentions to purchase online health popular science information. The results are shown in Table 7. As a result of the main effects test, we can conclude that perceived susceptibility weakens the negative implication between perceived health popular science information risk and online health popular science information purchase intention (β = 0.165, *p* < 0.001), and H6a was supported. And perceived susceptibility weakens the negative implication between perceived price unreasonableness and online health popular science information purchase intention (β = 0.161, *p* < 0.001); H6b was supported. Perceived severity weakened the negative implication between perceived health popular science information risk and purchase intention for online health popular science information (β = 0.183, *p* < 0.001); H7a was supported. Perceived severity weakened the negative implication between perceived price unreasonableness and the intention to purchase online health popular science information (β = 0.162, *p* < 0.001); H7b was supported.

## 6. Discussion

### 6.1. Key Findings

There are several major findings in this study. First, two health belief variables, perceived susceptibility and perceived severity, can significantly and positively influence users’ intentions to purchase online health popular science information. When users perceive that the health problems involved in online health popular science information are more likely to be contracted or have serious consequences, they are further motivated to purchase the information online. Most of the previous studies involving perceived susceptibility and perceived severity are based on a specific disease, exploring the effects of perceived susceptibility and perceived severity on disease-related preventive behaviors [75]. Moreover, in the field of online health, existing studies have mainly focused on online health information-seeking and -sharing behaviors, whereas few have focused on paid health information on the Internet. Based on the HBM and the characteristics of health popular science information on the Internet, we found that the perceived health popular science information risk significantly and negatively influenced users’ intentions to purchase online. When users have doubts about the credibility or source of online health popular science information and are not sure whether it is worth buying, the perceived risk of health popular science information is stronger, thus inhibiting their intention to buy online health popular science information. While scholars have mainly applied perceived information risk to the field of e-commerce, perceived information risk was found to be one of the most important factors influencing consumption decisions in the area of e-commerce [53]. This study applies information risk to an online health topic and finds a negative effect of perceived health information risk on purchase intention.

Second, based on the characteristics of paid content, we extracted two more variables, perceived irreplaceability and perceived price unreasonableness. Studies have proposed perceived irreplaceability and explored the drivers of this variable [68]. Perceived irreplaceability has also been found to have a significant positive effect on intention to adopt medical wearable technology [76]. And unlike previous studies, this paper explores the effect of perceived irreplaceability on the intention to purchase online health popular science information. This influence path has not yet been studied by scholars in related fields. In this study, perceived irreplaceability is applied to the field of paying for online health popular science information, and a positive relationship is found to influence the intention to purchase online health popular science information. It is found that perceived irreplaceability significantly and positively influences users’ intentions to purchase online health popular science information. Since there is a large amount of free health popular science information on the Internet, users are only motivated to pay for online health popular science information if they perceive a unique value from the paid information that is not available from other free sources. In addition, price is also a factor that cannot be ignored. If users think the price of online health popular science information is unreasonable and does not match the perceived value, it will weaken the users’ intentions to purchase online.

Finally, the moderating effects of perceived susceptibility and perceived severity in this study model were further explored. Most of the existing studies have explored the influence mechanisms of health beliefs such as perceived susceptibility and perceived severity as independent variables. In terms of moderating effects, some scholars have found that perceived susceptibility can significantly moderate the effects of perceived benefits and self-efficacy on safety behaviors [77]. Meanwhile, other scholars have found that increased susceptibility to COVID-19 can reduce the gap between physical activity intentions and behaviors [78]. In this present study, we found that both perceived susceptibility and perceived severity significantly attenuated the negative implication between perceived health popular science information risk and perceived price unreasonableness in online purchase intention. In other words, when users perceive that the health problems described in the online health popular science information are more likely to occur or have more serious consequences, users’ demand for this health popular science information will be stronger. And they will be more eager to learn how to avoid contracting such diseases. Therefore, the proportion of users’ doubts about the health popular science information itself and the consideration of price will be relatively lower.

### 6.2. Theoretical Contributions

This study provides the following main theoretical contributions:

(1) This study identifies the mechanisms by which perceived susceptibility and perceived severity affect the intention to purchase online health popular science information, expanding the application of the health belief model. Studies have mainly applied perceived susceptibility and perceived severity to online health information-seeking or -sharing, health-related behaviors on the Internet [32,33], or to offline health-related behaviors such as the intention to vaccinate [40,41]. The findings of existing studies have mainly found the positive influence of perceived susceptibility and perceived severity on online health information-seeking and offline disease-related prevention behaviors. In contrast, this study found a positive influence mechanism in perceived susceptibility and perceived severity on users’ online information purchasing behaviors such as purchasing online health popular science information. However, no studies have applied the above two health belief variables to an emerging online health behavior such as online health information payment. Moreover, most of the existing studies involving health belief variables discuss their effects on health behaviors as independent variables [51]; few studies have explored the moderating utility of health belief variables in online health information-related research. This study further explored the moderating effects of perceived susceptibility and perceived severity in the study of factors influencing intention to purchase health information, and it found that both can weaken the negative effects of perceived health popular science information risk and perceived price unreasonableness on purchase intention, which further expands the research related to intention to purchase online health information;

(2) This paper finds a negative relationship between perceived health popular science information risk and purchase intention for online health popular science information, enriching the research related to the influence mechanism of perceived information risk. The existing studies have mainly verified that perceived product information risk is one of the key factors influencing product purchase intention from the e-commerce domain [52,53]. In contrast, most of the current studies related to health behavior involve risks focusing on perceived disease risk. Perceived disease risk has been shown to significantly stimulate patients’ intentions to engage in health-related behaviors [79]. This paper combines the two, based on the special research scenario of paid online health popular science information and analyzes the impact of perceived health popular science information risk on the intention to purchase health popular science information from the perspectives of source credibility as well as perceived value, which establishes a new way of thinking for future research;

(3) In this paper, we found a positive correlation between perceived irreplaceability and the purchase intention for online health popular science information. Previous studies have proposed perceived irreplaceability and analyzed the drivers of perceived irreplaceability, i.e., proposing that perceived enjoyment and perceived usefulness are positively correlated with perceived irreplaceability [55,70]. There are also studies that have found a positive impact of perceived irreplaceability on the acceptance of medical wearables [78], whereas it is currently unknown what effect perceived irreplaceability as an antecedent variable will have on intention to purchase online health popular science information. This paper explores the effect of perceived irreplaceability on the intention to purchase online health popular science information and verifies this positive influence mechanism, further enriching the related research on the influence mechanism of perceived irreplaceability.

### 6.3. Practical Contribution

This study provides the following main practical contributions:

(1) The results of this study have important implications for creators of online health popular science information. The creators can write the information based on the conclusions of this study. For example, the creators should emphasize the uniqueness of the content, the importance of the health issues involved, and set a reasonable price when publishing paid health popular science information. They can also list the sources of the professional content or the creators’ own professional qualifications as much as possible to gain the trust of users. In addition, the publisher can target the public popularization of recent concerns about health in popular issues according to the different stages of time and different regions of the epidemic, to further attract readers’ interest in reading and thus generate the intention to buy. All these measures can increase the purchase of online health popular science information;

(2) This study can provide an important reference for users to purchase better quality online health popular science information. For example, users can perceive the value of the limited information they see and judge whether they can obtain it from free health popular science information. They can also measure the perceived risk of health popular science information, based on the professional qualifications of the creator, the reference source of the information, and the value of the purchase;

(3) This study also has important implications for the sustainability of the platform. The platform can push the popular health problems in the user’s location according to the IP address of different users, in order to attract the attention of the user to stimulate their purchase intention. Users purchase online health popular science information, and the platform can gain corresponding revenue. Therefore, the platform can further optimize the paid segments based on the findings of this paper. For example, the platform can focus on recommending health popular science information that involves high susceptibility and severity in health problems. Platforms can require creators to introduce their relevant professional qualifications as comprehensively as possible, in order to obtain higher revenue and promote the prosperity of the platform.

### 6.4. Limitations and Prospects

(1) The sample data size used in this study is limited, and the sample subjects are relatively concentrated in terms of education and occupation, covering insufficient groups. Therefore, the sample size can be further increased in the future to collect groups with different education and occupations to further test the research hypothesis. (2) No study has focused on paid health popular science information and explored the factors influencing users’ intentions to purchase. Instead, the findings of this study explore the influencing factors in purchase intention only from the perspective of health belief modeling. There are many factors affecting the intention to buy, and future research can further expand the research related to the factors influencing the intention to buy online health popular science information from other dimensions. (3) Online health knowledge payment is a new research field. There have been studies focusing mainly on a knowledge-based payment course, whereas there are yet no studies that have focused on paid health popular science information on the Internet. This paper only focuses on the intention to purchase paid health information on online social platforms in China. In the future, we can continue to pay attention to the progress of research related to online health knowledge payment in different countries, regions, and time periods and compare it with the findings of this study.

## 7. Conclusions

This study applied the health belief model (HBM) to the scenario of online paid health popular science information. And this paper introduced three variables, including perceived health popular science information risk, perceived irreplaceability, and perceived price unreasonableness based on the characteristics of online paid health popular science information. The results show that two health belief variables, perceived susceptibility and perceived severity, positively influence users’ intentions to purchase health popular science information on the Internet. Online health popular science information stimulates users’ purchase intentions when it involves health problems that are more susceptible to infection or have more serious consequences of infection. Perceived irreplaceability positively influences users’ intentions to purchase online. When users perceive a unique value from the limited content of paid online health popular science information, it will further push them to purchase. Perceived health popular science information risks negatively influencing users’ intentions to purchase online. When users have doubts about the source or credibility of online health popular science information, users perceive more risks in online health popular science information. This feeling will inhibit their intentions to purchase. Perceived price unreasonableness negatively affects users’ intentions to purchase online health popular science information. When users believe that the pricing of health popular science information does not match the perceived value, they will think that the price setting is unreasonable. And their intention to buy online health popular science information will be weakened.

Moreover, this study further explored the moderating effect of health belief variables. The results showed that both perceived susceptibility and perceived severity could weaken the negative implication of perceived health popular science information risk and perceived price unreasonableness on users’ online purchase intentions. When users perceive that the health problems mentioned in the health popular science information are more susceptible or more serious, they have a stronger need to know the corresponding countermeasures for such health problems. At this time, the consideration of the risks of health popular science information and the reasonableness of price setting will be relatively reduced. The findings of this study are important for the creators of health popular science information. These findings could help them to write health popular science information that is of more interest to the public. Based on the findings of this study, publishers can write online health popular science information that is more popular among users thus prompting their purchase intentions. Users who receive high quality health information will have a positive impact on their own health, which in turn will improve public health as a whole. At the same time, our findings also have important reference values for the management of the paid health content of the platform. This study can promote the prosperity of the platform.

## Figures and Tables

**Table 1:** Questionnaire variables, question items, and sources.

Variables	Name	Items	Sources
Perceived susceptibility	PSU1	The health problems mentioned in the online health popular science information are likely to occur to me	[46]
	PSU2	I am likely to encounter the health problems mentioned in the online health popular science information	
	PSU3	I am likely to contract the health-related problems mentioned in the online health popular science information	
Perceived severity	PSE1	The health problems mentioned in the online health popular science information have serious consequences for me	[40]
	PSE2	The health issues mentioned in the online health popular science information on infection can have a significant impact on me	
	PSE3	Having the disease mentioned in the online health popular science information is a serious problem for me	
Perceive health popular science information risks	PHIR1	I have doubts about the credibility of online health popular science information	[21,67]
	PHIR2	I am concerned that online health popular science information may have a negative impact on my health	
	PHIR3	I have doubts about the source of online health popular science information	
	PHIR4	I am not sure if online health popular science information is worth buying	
Perceived irreplaceability	PIRE1	I am finding it hard to find other free online health popular science information that provides the same value	[68]
	PIRE2	I had a hard time finding other free online health popular science information to replace it	
	PIRE3	I cannot easily replace the paid online health popular science information with other information	
Perceived price unreasonableness	PPUN1	I think the cost of online health popular science information is higher	[69]
	PPUN2	I think the price set for online health popular science information is unreasonable	
	PPUN3	I cannot receive the current rates for online health popular science information	
Purchase intention for online health popular science information	PI1	I am willing to pay to access the content in online health popular science information	[70]
	PI2	I would consider purchasing online health popular science information	

**Table 2:** Demographic data.

Demographic Characteristics		Frequency	Percentage
Gender	Male	259	42.88
	Female	345	57.12
Age	<18	4	0.66
	19–25	110	18.21
	26–30	181	29.97
	31–40	261	43.21
	41–50	31	5.13
	51–60	16	2.65
	>61	1	0.17
Education	High school or below	18	2.98
	Specialized school	54	8.94
	Undergraduate	458	75.83
	Master’s or above	74	12.25
Income	<1000	22	3.64
	1001–3000	49	8.11
	3001–5000	57	9.44
	5001–8000	195	32.28
	8001–15,000	179	29.64
	>15,000	102	16.89
Occupation	Students	73	12.09
	State agencies, institutions	84	13.91
	Corporate staff	420	69.54
	Other occupations	27	4.47
Current city of residence	Level 1	275	45.53
	Level 2	147	24.34
	Level 3	92	15.23
	Level 4	69	11.42
	Level 5	21	3.48

**Table 3:** Results of reliability and aggregate validity.

Variable	Factor	Factor Loading	Cronbach’s Alpha	CR	AVE
Perceived susceptibility	PSU1	0.844	0.711	0.838	0.634
	PSU2	0.708			
	PSU3	0.831			
Perceived severity	PSE1	0.891	0.841	0.904	0.759
	PSE2	0.847			
	PSE3	0.874			
Perceive health popular science information risks	PHIR1	0.871	0.872	0.912	0.721
	PHIR2	0.772			
	PHIR3	0.877			
	PHIR4	0.872			
Perceived irreplaceability	PIRE1	0.919	0.863	0.914	0.781
	PIRE2	0.913			
	PIRE3	0.814			
Perceived price unreasonableness	PPUN1	0.872	0.866	0.918	0.788
	PPUN2	0.886			
	PPUN3	0.905			
Purchase intention for online health popular science information	PI1	0.896	0.734	0.882	0.790
	PI2	0.881			

**Table 4:** Correlation results of the scale.

	PI	PIRE	PHIR	PSE	PSU	PPUN
**PI**	**0.889**					
**PIRE**	0.296	**0.884**				
**PHIR**	−0.451	−0.196	**0.849**			
**PSE**	0.280	0.361	−0.214	**0.871**		
**PSU**	0.254	0.256	−0.208	0.274	**0.797**	
**PPUN**	−0.447	−0.176	0.721	−0.185	−0.178	**0.888**

**Table 5:** Regression results of control variables PLS.

Path	β	T-Statistic	*p*-Value
Current place of residence -> PI	0.044	1.072	0.284
Age -> PI	0.039	0.831	0.406
Education -> PI	0.001	0.011	0.991
Gender -> PI	0.145 ***	3.839	**0.000**
Income -> PI	0.217 ***	4.230	**0.000**

Note: *** *p* < 0.001.

**Table 6:** Main effect result test.

Hypothesis	Path	β	T-Statistic	Supported?
H1	PSU -> PI	0.101 *	2.395	Supported
H2	PSE -> PI	0.112 *	2.322	Supported
H3	PHIR -> PI	−0.211 ***	3.490	Supported
H4	PIRE -> PI	0.148 ***	3.710	Supported
H5	PPUN -> PI	−0.231 ***	3.734	Supported

Note: * *p* < 0.05; *** *p* < 0.001.

**Table 7:** Test of moderating effect results.

Hypothesis	Path	β	T-Statistic	Supported?
H6a	PHIR × PSU -> PI	0.165 ***	3.631	Supported
H6b	PPUN × PSU -> PI	0.161 ***	3.656	Supported
H7a	PHIR × PSE -> PI	0.183 ***	3.592	Supported
H7b	PPUN × PSE -> PI	0.162 **	2.855	Supported

Note: ** *p* < 0.01; *** *p* < 0.001.

## Data Availability

Data sharing is not applicable to this article, due to privacy and ethical restrictions.

## Abbreviations

The following abbreviations are used in this manuscript:

OHC	Online health community
HBM	Health belief model
PSU	Perceived susceptibility
PSE	Perceived severity
PHIR	Perceived health popular science information risk
PIRE	Perceived irreplaceability
PPUN	Perceived price unreasonableness
PI	Purchase intention for online health popular science information
PLS	Partial least squares
CR	Composite reliability
AVE	Average variance extracted

## References

[1] Su L., Li Y., Li W. (2019). Understanding Consumers’ Purchase Intention for Online Paid Knowledge: A Customer Value Perspective. Sustainability.

[2] Zhang S., Wang W.T., Wang Y.Z. Research on the Influencing Factors of User’s Online Knowledge Consumption Behavior. Proceedings of the 5th International Conference on Information Technology and Quantitative Management (ITQM).

[3] Weiwei Z., Ling Z. (2023). The effect of textual features of paid-for health knowledge product descriptions on purchase volume. Libr. Trib..

[4] Yang Y., Zhu X., Song R., Zhang X., Guo F. (2021). Not just for the money? An examination of the motives behind physicians’ sharing of paid health information. J. Inf. Sci..

[5] Parkinson J., Adendorff R. (2004). The use of popular science articles in teaching scientific literacy. Engl. Specif. Purp..

[6] Xiao N., Sharman R., Rao H.R., Upadhyaya S. (2014). Factors influencing online health information search: An empirical analysis of a national cancer-related survey. Decis. Support Syst..

[7] Hsu W.C. (2021). Undergraduate Students’ Online Health Information-Seeking Behavior during the COVID-19 Pandemic. Int. J. Environ. Res. Public Health.

[8] Chen Y.Y., Li C.M., Liang J.C., Tsai C.C. (2018). Health Information Obtained from the Internet and Changes in Medical Decision Making: Questionnaire Development and Cross-Sectional Survey. J. Med. Internet Res..

[9] Lederman R., Fan H.M., Smith S., Chang S.T. (2014). Who can you trust? Credibility assessment in online health forums. Health Policy Technol..

[10] Qi T., Wang T., Yan J. (2021). The spillover effects of different monetary incentive levels on health experts’ free knowledge contribution behavior. Internet Res..

[11] Jiang W., Lusha Z. (2017). A Study on the Influencing Factors of Purchasing Decisions of Internet Health Information Service Users. J. China Soc. Sci. Tech. Inf..

[12] Shun C., Hairong S., Xin F., Xi C. (2019). Study on the Influencing Factors of Knowledge Payment Product Sales: Taking Zhihu Live as an Example. J. Ind. Eng. Eng. Manag..

[13] Meei-Shia C., Land K.C. (1986). Testing the health belief model: LISREL analysis of alternative models of causal relationships between health beliefs and preventive dental behavior. Soc. Psychol. Q.

[14] Shi X., Zheng X., Yang F. (2020). Exploring payment behavior for live courses in social Q&A communities: An information foraging perspective. Inf. Process. Manag..

[15] Zhao Y., Zhao Y., Yuan X., Zhou R. (2018). How knowledge contributor characteristics and reputation affect user payment decision in paid Q&A? An empirical analysis from the perspective of trust theory. Electron. Commer. Res. Appl..

[16] Mannan M., Ahamed R., Zaman S.B. (2019). Consumers’ willingness to purchase online mental health services. J. Serv. Mark..

[17] Zhang J., Zhang J., Zhang M. (2019). From free to paid: Customer expertise and customer satisfaction on knowledge payment platforms. Decis. Support Syst..

[18] Daradkeh M., Gawanmeh A., Mansoor W. (2022). Information Adoption Patterns and Online Knowledge Payment Behavior: The Moderating Role of Product Type. Information.

[19] Zhao Y., Liu Z., Song S. (2018). Why Should I Pay for the Knowledge in Social Q&A Platforms?. Transform. Digit. Worlds.

[20] Yu L., Chen Z., Yao P., Liu H. (2021). A Study on the Factors Influencing Users’ Online Knowledge Paying-Behavior Based on the UTAUT Model. J. Theor. Appl. Electron. Commer. Res..

[21] Xu A., Li W., Chen Z., Zeng S., Carlos L.-A., Zhu Y. (2021). A Study of Young Chinese Intentions to Purchase “Online Paid Knowledge”: An Extended Technological Acceptance Model. Front Psychol..

[22] Vahabzadeh A., Wittenauer J., Carr E. (2011). Stigma, Schizophrenia, and the Media: Exploring Changes in the Reporting of Schizophrenia in Major US Newspapers. J. Psychiatr. Pract..

[23] Ameri F., Keeling K., Salehnejad R. (2020). You Get What You Pay for on Health Care Question and Answer Platforms: Nonparticipant Observational Study. J. Med. Internet Res..

[24] Meng F., Zhang X., Liu L., Ren C. (2021). Converting readers to patients? From free to paid knowledge-sharing in online health communities. Inf. Process. Manag..

[25] Wu B., Luo P., Li M., Hu X. (2022). The Impact of Health Information Privacy Concerns on Engagement and Payment Behaviors in Online Health Communities. Front. Psychol..

[26] Fiedler M., Sarstedt M. (2014). Influence of community design on user behaviors in online communities. J. Bus. Res..

[27] Rutten L.J.F., Blake K.D., Greenberg-Worisek A.J., Allen S.V., Moser R.P., Hesse B.W. (2019). Online Health Information Seeking Among US Adults: Measuring Progress Toward a Healthy People 2020 Objective. Public Health Rep..

[28] Tao D., Wang T., Wang T., Zhang T., Zhang X., Qu X. (2020). A systematic review and meta-analysis of user acceptance of consumer-oriented health information technologies. Comput. Hum. Behav..

[29] del Mar García-de los Salmones M., Herrero A., Martínez P. (2020). Determinants of Electronic Word-of-Mouth on Social Networking Sites about Negative News on CSR. J. Bus. Ethics.

[30] Anastasiei B., Dospinescu N., Dospinescu O. (2023). Word-of-Mouth Engagement in Online Social Networks: Influence of Network Centrality and Density. Electronics.

[31] Janz N.K., Becker M.H. (1984). The Health Belief Model: A decade later. Health Educ. Q..

[32] Wong L.P., Alias H., Wong P.-F., Lee H.Y., Abubakar S. (2020). The use of the health belief model to assess predictors of intent to receive the COVID-19 vaccine and willingness to pay. Hum. Vaccine Immunother..

[33] Zampetakis L.A., Melas C. (2021). The health belief model predicts vaccination intentions against COVID-19: A survey experiment approach. Appl. Psychol. Health Well Being.

[34] Sumita I., Toyama N., Ekuni D., Maruyama T., Yokoi A., Fukuhara D., Uchida-Fukuhara Y., Nakahara M., Morita M. (2022). The Impact of Oral Health Behaviors, Health Belief Model, and Absolute Risk Aversion on the Willingness of Japanese University Students to Undergo Regular Dental Check-Ups: A Cross-Sectional Study. Int. J. Environ. Res. Public Health.

[35] Kim S.C., Cooke S.L. (2020). Using the Health Belief Model to Explore the Impact of Environmental Empathy on Behavioral Intentions to Protect Ocean Health. Environ. Behav..

[36] Kim N., Lee S., Lee C.-K., Suess C. (2022). Predicting preventive travel behaviors under the COVID-19 pandemic through an integration of Health Belief Model and Value-Belief-Norm. Tour. Manag. Perspex..

[37] Huang X., Dai S., Xu H. (2020). Predicting tourists’ health risk preventative behavior and travelling satisfaction in Tibet: Combining the theory of planned behavior and health belief model. Tour. Manag. Perspect..

[38] Zhao J., An Y. (2021). Behavioral intention of forest therapy tourism in China: Based on health belief model and the theory of planned behavior. Curr. Issues Tour..

[39] Ghorbani-Dehbalaei M., Loripoor M., Nasirzadeh M. (2021). The role of health beliefs and health literacy in women’s health promoting behaviors based on the health belief model: A descriptive study. BMC Womens Health.

[40] Shang L., Zhou J., Zuo M. (2020). Understanding older adults’ intention to share health information on social media: The role of health belief and information processing. Internet Res..

[41] Zhao Y.C., Zhao M., Song S. (2022). Online Health Information Seeking Among Patients with Chronic Conditions: Integrating the Health Belief Model and Social Support Theory. J. Med. Internet Res..

[42] Zhao H., Deng S., Liu Y., Xia S., Lim E.T.K., Tan C.-W. (2022). Promoting users’ smartphone avoidance intention: The role of health beliefs. Ind. Manag. Data Syst..

[43] Wang Y., Wu A.M.S., Lau J.T.F. (2016). The health belief model and number of peers with internet addiction as inter-related factors of Internet addiction among secondary school students in Hong Kong. BMC Public Health.

[44] Glanz K., Rimer B.K., Viswanath K. (2008). Health behavior and health education: Theory, research, and practice. Oncol. Nurs. Forum.

[45] Maiman L.A., Becker M.H. (1974). The health belief model: Origins and correlates in psychological theory. Health Educ. Monogr..

[46] Ahadzadeh A.S., Sharif S.P., Ong F.S., Khong K.W. (2015). Integrating health belief model and technology acceptance model: An investigation of health-related internet use. J. Med. Internet Res..

[47] Mou J., Shin D.-H., Cohen J. (2016). Health beliefs and the valence framework in health information seeking behaviors. Inf. Technol. People.

[48] Nong Y., Zhao M., Chien H. (2022). Path relationship of consumers’ perceived susceptibility and severity of health problems with their purchase of buckwheat functional foods in China. Heliyon.

[49] Lee K.-Y., Wei C.-Y., Wu M.-H., Hsieh C.-M. (2020). Determinants of the Public Health Promotion Behavior: Evidence from Repurchasing Health Foods for Improving Gastrointestinal Tract Functions. Int. J. Environ. Res. Public Health.

[50] Park S., Oh S. (2022). Factors associated with preventive behaviors for COVID-19 among adolescents in South Korea. J. Pediatr. Nurs.-Nurs. Care Child. Fam..

[51] Mirakzadeh A.A., Karamian F., Khosravi E., Parvin F. (2021). Analysis of Preventive Behaviors of Rural Tourism Hosts in the Face of COVID-19 Pandemic: Application of Health Belief Model. Front. Public Health.

[52] Rodgers W.H. (1969). Consumer Behavior as Risk Taking.

[53] Chiu C., Wang E.T.G., Fang Y., Huang H. (2014). Understanding customers’ repeat purchase intentions in B2C e-commerce: The roles of utilitarian value, hedonic value, and perceived risk. Inf. Syst. J..

[54] Shah Z., Wei L. (2022). Source Credibility and the Information Quality Matter in Public Engagement on Social Networking Sites during the COVID-19 Crisis. Front. Psychol..

[55] Grau C. (2004). Irreplaceability and Unique Value. Philos. Top..

[56] Young K.S. (1999). Internet addiction: Evaluation and treatment. Br. Med. J. Publ. Group.

[57] Hsiao K.-L., Chen C.-C. (2017). Value-based adoption of e-book subscription services: The roles of environmental concerns and reading habits. Telemat. Inform..

[58] Wang Y.-S., Yeh C.-H., Liao Y.-W. (2013). What drives purchase intention in the context of online content services? The moderating role of ethical self-efficacy for online piracy. Int. J. Inf. Manag..

[59] Anselmsson J., Burt S., Tunca B. (2017). An integrated retailer image and brand equity framework: Re-examining, extending, and restructuring retailer brand equity. J. Retail. Consum. Serv..

[60] Leung X.Y., Cai R. (2021). How pandemic severity moderates digital food ordering risks during COVID-19: An application of prospect theory and risk perception framework. J. Hosp. Tour. Manag..

[61] Kim D.H. (2019). How do you feel about a disease? The effect of psychological distance towards a disease on health communication. Int. J. Advert..

[62] Zheng L., Miao M., Gan Y. (2020). Perceived Control Buffers the Effects of the COVID-19 Pandemic on General Health and Life Satisfaction: The Mediating Role of Psychological Distance. Appl. Psychol.-Health Well Being.

[63] Zheng L., Miao M., Lim J., Li M., Nie S., Zhang X. (2020). Is lockdown bad for social anxiety in COVID-19 regions? A national study in the SOR perspective. Int. J. Environ. Res. Public Health.

[64] Cai R., Leung X.Y. (2020). Mindset matters in purchasing online food deliveries during the pandemic: The application of construal level and regulatory focus theories. Int. J. Hosp. Manag..

[65] Updegraff J.A., Brick C., Emanuel A.S., Mintzer R.E., Sherman D.K. (2015). Message framing for health: Moderation by perceived susceptibility and motivational orientation in a diverse sample of Americans. Health Psychol..

[66] Liu J., Lu S., Lu C. (2022). What Motivates People to Receive Continuous COVID-19 Vaccine Booster Shots? An Expectation Confirmation Theory Perspective. Healthcare.

[67] Wasiuzzaman S. (2021). Regulations, perceived information quality and perceived risk of equity crowdfunding: A study of Malaysianinvestors. Strateg. Change.

[68] Wang C., Lee M.K., Hua Z. (2015). A theory of social media dependence: Evidence from microblog users. Decis. Support Syst..

[69] Kuo Y.-F., Yen S.-N. (2009). Towards an understanding of the behavioral intention to use 3G mobile value-added services. Comput. Hum. Behav..

[70] Chen Y., Ding D., Meng L., Li X., Zhang S. (2021). Understanding consumers’ purchase intention towards online paid courses. Inf. Dev..

[71] Hair J.F., Sarstedt M., Ringle C.M., Mena J.A. (2012). An assessment of the use of partial least squares structural equation modeling in marketing research. J. Acad. Mark. Sci..

[72] Fornell C., Larcker D.F. (1981). Evaluating structural equation models with unobservable variables and measurement error. J. Mark. Res..

[73] Gefen D., Straub D. (2005). A practical guide to factorial validity using PLS-Graph: Tutorial and annotated example. Commun. Assoc. Inf. Syst..

[74] Hair J.F., Ringle C.M., Sarstedt M. (2011). PLS-SEM: Indeed, a silver bullet. J. Mark. Theory Pract..

[75] An S., Schulz P.J., Kang H. (2023). Perceived COVID-19 susceptibility and preventive behaviors: Moderating effects of social support in Italy and South Korea. BMC Public Health.

[76] Zhang M., Luo M., Nie R., Zhang Y. (2017). Technical attributes, health attribute, consumer attributes and their roles in adoption intention of healthcare wearable technology. Int. J. Med. Inform..

[77] Ng B.-Y., Kankanhalli A., Xu Y. (2009). Studying users’ computer security behavior: A health belief perspective. Decis. Support Syst..

[78] Ahn J., Kim Y., Jang D. (2023). Physical activity intention-behavior gap during the COVID-19 pandemic: The moderating role of motivation and health-belief. Int. J. Sport Exerc. Psychol..

[79] Shiloh S., Deheer H., Peleg S., Alford S.H., Skapinsky K., Roberts J., Hadley D. (2015). The impact of multiplex genetic testing on disease risk perceptions. Clin. Genet..

